# Use of low-dose immune checkpoint inhibitors in low- and middle-income countries: a national survey of oncologists and hematologists in Mexico

**DOI:** 10.3332/ecancer.2026.2144

**Published:** 2026-06-10

**Authors:** Andres Meraz-Brenez, Enrique Soto-Perez-De-Celis, Roberta Demichelis-Gomez, Leonardo Verduzco-Rodriguez, Arjun Gupta, Meera Ragavan, Fumiko Chino, Haydee Cristina Verduzco-Aguirre

**Affiliations:** 1 Department of Medical Oncology, Hospital de Oncología, Centro Médico Nacional Siglo XXI, Instituto Mexicano del Seguro Social, Mexico City 06725, Mexico; 2 University of Colorado Anschutz Medical Campus, Aurora, CO 80045, USA; 3 Instituto Nacional de Ciencias Médicas y Nutrición Salvador Zubirán, Mexico City 14080, Mexico; 4 Hospital Regional de Rio Blanco, Rio Blanco 94733, Mexico; 5 M Health Fairview Clinics and Surgery Center, Minneapolis, MN 55455, USA; 6 Costs of Care, Boston, MA 02139, USA; 7 Kaiser Permanente San Francisco Medical Center, San Francisco, CA 94115, USA; 8 MD Anderson Cancer Center, Houston, TX 77030, USA

**Keywords:** immune checkpoint inhibitors, drug costs, Mexico, neoplasms/drug therapy

## Abstract

Immune checkpoint inhibitors (ICIs) have transformed the treatment landscape for multiple cancers but remain largely inaccessible in low- and middle-income countries (LMICs) due to high costs and limited availability. Pharmacokinetic and pharmacodynamic data, together with emerging clinical evidence, suggest that standard approved doses may exceed the threshold required for therapeutic efficacy, supporting the biological plausibility of low-dose strategies. In LMICs, such approaches could broaden access, yet their adoption is still limited. Eighty-one Mexican oncologists and hematologists answered a web-based survey designed to examine the use of low-dose ICI, reasons for implementation and barriers to adoption. All respondents reported at least one barrier to prescribing ICI, most commonly economic limitations or lack of coverage (71.6%). Nearly half (45.7%) reported having treated at least one patient with low-dose ICI, most often with the intention of reducing treatment costs (75.7%). Nivolumab was the most frequently used drug in low-dose regimens (75.7%), followed by pembrolizumab (43.2%). Commonly perceived barriers to prescribing low-dose ICI included concerns about efficacy and safety (51.9%), insufficient knowledge of dosing protocols (51.9%) and insurance approval limitations (43.2%). Confidence in efficacy was higher in the palliative compared to the curative setting. Only 19.7% of respondents felt sufficient evidence and resources were available to support the implementation of low-dose ICI. This study provides evidence on the use of low-dose ICI among physicians in Mexico, reflecting strategies motivated by cost reduction but hindered by safety concerns and perceived lack of evidence. As access to ICI remains a significant challenge in LMICs, the rigorous evaluation and implementation of low-dose regimens should be considered a research and policy priority.

## Introduction

Immune checkpoint inhibitors (ICIs) have transformed the treatment landscape for a wide range of malignancies, achieving durable responses and survival benefits in settings where conventional therapies have limited impact [1, 2]. However, their high cost and limited availability represent barriers to access, particularly in low- and middle-income countries (LMICs) [3]. Economic and structural inequalities, along with high out-of-pocket and indirect costs faced by patients, contribute to the financial toxicity of cancer care [4]. 

In Mexico, an upper-middle-income country, most patients with cancer receive treatment in the public health sector, through either contributive social security or the federal and/or state ministries of health [5]. Despite recent reforms aimed at improving access to innovative cancer medicines, this remains unequal and intermittent across financing schemes and regions [6]. While ICI has been included in public formularies across various LMICs, coverage often remains limited to selected indications or institutions. Combined with the lack of universal coverage and high direct household health spending, this means that only a minority of eligible patients receive these therapies, often through private insurance [7].

Emerging pharmacokinetic and pharmacodynamic data suggest that standard-approved ICI doses may substantially exceed the threshold required for therapeutic efficacy [8]. Early-phase trials and modeling analyses indicate a non-linear dose–response curve for agents such as nivolumab and pembrolizumab, in which dose reductions within a certain range do not appear to compromise antitumour activity [8]. These findings, combined with the long half-life and high binding affinity of monoclonal antibodies, provide a biologically plausible rationale for exploring low-dose strategies. Clinical evidence across several tumour types, including head and neck squamous carcinoma (HNSCC), microsatellite instability-high colon cancer and lung cancer, indicates that reduced ICI doses achieve clinical outcomes comparable to standard dose regimens and supports further exploration of such strategies [9–11].

In LMICs, low-dose ICI regimens could have practical and transformative implications by supporting a pathway to broader access. However, the adoption of low-dose regimens remains limited by the lack of robust evidence for their use, the absence of clinical guidelines endorsing alternative dosing, regulatory restrictions, legal considerations and physician perceptions and willingness to deviate from label-recommended doses.

Understanding how physicians approach and perceive low-dose ICI use is essential to inform future clinical guidance and policy. To address this gap, we conducted a national survey of medical oncologists and hematologists in Mexico to assess the adoption of low-dose ICI regimens in routine clinical practice, as well as perceptions and barriers regarding their use.

## Methods

### Study design and participants

This was a cross-sectional, web-based survey of medical oncologists and hematologists actively practicing in Mexico. The survey was distributed between April and May 2024, with bi-weekly reminders sent through the email lists of the Mexican Society of Oncology (Sociedad Mexicana de Oncologí) and the Mexican Group for the Study of Hematology (Agrupación Mexicana para el Estudio de la Hematología), which include approximately 2,000 physicians. The survey was also disseminated using snowball sampling through physician-led oncology and hematology WhatsApp groups. Responses were collected using REDCap electronic data capture tools [12].

### Survey instrument

The survey instrument was developed based on a literature review and investigator consensus and consisted of 22 structured, closed-ended questions (translated survey available in the Appendix). Collected data included demographic information (age, gender, specialty, years in practice and type of practice setting), access to ICI, perceived barriers to access and current prescribing patterns, including use of low-dose ICI. Low-dose ICI was defined as any regimen administered at any dose below the one recommended in the prescribing information for each medication approved in Mexico by the Federal Commission for the Protection against Sanitary Risk (Comisión Federal para la Protección contra Riesgos Sanitarios), the national regulatory agency.

Respondents were asked to report on the agents used in low-dose regimens and perceived advantages of this approach. In addition, the survey assessed physician confidence in the efficacy and safety of low-dose ICI for both the curative and palliative setting, as well as perceived barriers to the adoption of this strategy. Questions on barriers, perceived advantages and potential risks of low-dose ICI allowed multiple selections.

### Outcomes

The primary outcome was the frequency of low-dose ICI use among survey respondents. Secondary outcomes included the identification of specific agents used in low-dose regimens; the main reasons for adopting low-dose strategies; physician-reported confidence in the efficacy of low-dose ICI by treatment intent (curative versus palliative); and perceived barriers to adoption.

### Statistical analysis

Analyses were performed using complete responses for each item. Descriptive analyses were performed using SPSS 25.0 (IBM Corp, Armonk, NY); proportions, mean values with standard deviations and medians with interquartile ranges (IQR) are reported as appropriate. We compared the frequency of low-dose ICI use according to practice setting and specialty using the chi-square test and differences in medians among groups using the Mann-Whitney *U* and Kruskal-Wallis tests; a *p*-value <0.05 was considered statistically significant.

### Ethics

This study was approved by the Institutional Review Board at Instituto Nacional de Ciencias Médicas y Nutrición Salvador Zubirán (approval number HEM-4955-24-24-1).

## Results

A total of 131 individuals started the survey. Of these, 81 (61.8%) answered the primary question about low-dose ICI use, while 50 exited the survey after completing the demographic section. Therefore, all analyses are based on the 81 complete responses. Since participants were recruited through snowball sampling, the total number of invited individuals could not be ascertained and a response rate could not be calculated.

Among the 81 respondents, 63 (77.8%) were medical oncologists and 18 (22.2%) were hematologists. Forty-six (56.8%) identified as male, 32 (39.5%) as female and two (2.5%) as non-binary. The mean age was 44.2 ± 11.1 years. Regarding practice setting, 23 respondents (28.4%) worked exclusively in private practice, 11 (13.6%) in public institutions and 47 (58.0%) in both, as shown in Table 1.

Fifty-eight participants (71.6%) reported difficulties with the procurement or availability of ICI in their practice. All respondents reported at least one barrier to prescribing ICI, most commonly economic limitations (71.6%), lack of coverage (71.6%), regulatory restrictions (43.2%) and limited drug availability (43.2%). Less frequently reported barriers included geographic limitations (6.2%) and other issues (3.7%), such as delayed insurance approval and administrative delays in drug procurement within the public sector. Respondents reported that the median proportion of patients paying out of pocket for ICI treatment is 15% (IQR 5–30), with statistically significant differences among types of practice (exclusively public 0% (IQR 0–10)], exclusively private 10% (IQR 5–25), both sectors 10% (IQR 5–30); *p* = 0.019) as well as between specialties (medical oncology 5% (IQR 0–20), hematology 20% (IQR 7.5–32.5); *p* = 0.028).

Thirty-seven respondents (45.7%) had treated at least one patient with low-dose ICI. No statistically significant difference in low-dose ICI use was observed by specialty (46.0% of medical oncologists versus 44.4% of hematologists; *p* = 0.90). Low-dose ICI use was less common among physicians practicing exclusively in the public setting (27.3%) than those in private practice (43.5%) or combined public-private practice (51.1%). These differences were not statistically significant (*p* = 0.35).

Among the 37 respondents who reported low-dose ICI use, the most common reason was to reduce treatment costs for patients (75.7%), followed by minimising drug waste through vial-sharing strategies (29.7%) and improving tolerability (27%). Two respondents reported using low-dose ICI in the context of a clinical trial. Nivolumab was the most frequently prescribed agent in low-dose regimens (75.7%), followed by pembrolizumab (43.2%) and ipilimumab (21.6%). Only one respondent reported using durvalumab (2.7%) and none reported using atezolizumab in low-dose regimens.

**Figure 1.**
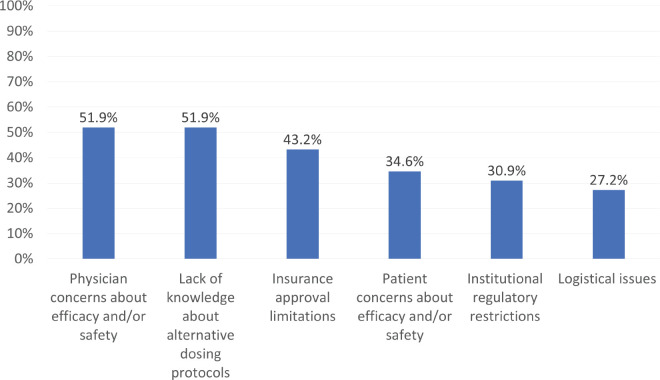
Reported barriers for the use of low-dose immunotherapy (N = 81).

Figure 1 shows the most frequently reported barriers to adopting low-dose ICI: concerns about efficacy and safety (51.9%), insufficient knowledge of alternative dosing protocols (51.9%) and insurance approval limitations (43.2%). Additional barriers included patient concerns regarding efficacy and safety (34.6%), institutional regulatory restrictions (30.9%) and logistical challenges such as scheduling for vial sharing (27.2%) (Figure 1).

**Figure 2.**
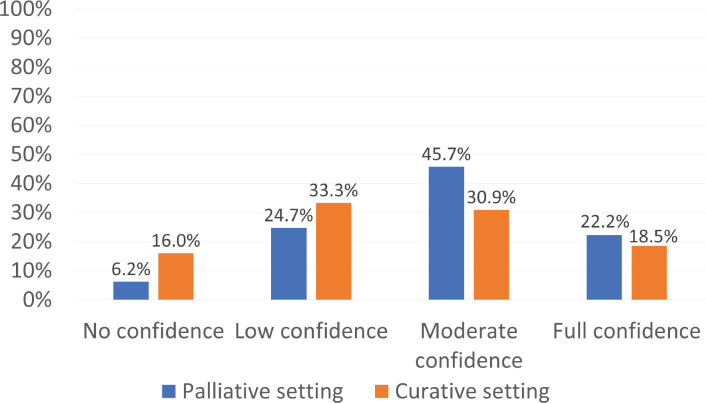
Physician confidence in the efficacy of low-dose immunotherapy regimens (N = 81).

Regarding confidence in the efficacy of low-dose ICI in the curative setting, 13 respondents (16.0%) reported no confidence, 27 (33.3%) had low confidence, 25 (30.9%) had moderate confidence and 15 (18.5%) expressed complete confidence. In the palliative setting, five respondents (6.2%) reported no confidence, 20 (24.7%) had low confidence, 30 (37.0%) had moderate confidence and 18 (22.2%) had complete confidence, as shown in Figure 2.

A minority of respondents (19.7%) considered that sufficient information and resources were available to support the implementation of low-dose ICI regimens. Perceived potential benefits included reducing the financial burden for patients (71.6%) and for the healthcare system (70.4%), followed by decreasing toxicity (44.4%) and increasing administration flexibility (33.3%). Three respondents (3.7%) did not perceive any benefits from low-dose ICI use.

## Discussion

In this national survey of Mexican oncologists and hematologists, nearly half of the respondents reported prescribing ICI at doses lower than those approved on the product label. The most common reason for low-dose ICI use was reducing treatment costs for patients. Concerns about efficacy and safety were common, with greater confidence in the efficacy of low-dose ICI in the palliative setting compared to the curative setting.

Participants viewed the primary advantage of low-dose ICI as reduced costs for both patients and the healthcare system, aligning with existing evidence that affordability is a crucial factor in accessing immunotherapy [13]. The higher proportion of physicians using low-dose ICI in private practice, although seemingly counterintuitive, may reflect that patients in this sector more often pay out-of-pocket for medications, as only about 10% of Mexicans have private health insurance [14]. This should not be interpreted as indicating that the remaining patients had full insurance coverage or unrestricted access to standard-dose therapy. In Mexico, access is frequently constrained by fragmented coverage, administrative approval requirements, intermittent availability in public systems and partial coverage. As a result, patients may experience delayed or limited access, receive modified dosing strategies or not receive immunotherapy at all.

For most Mexicans, paying out-of-pocket for ICI is financially unfeasible. A 40 mg vial of nivolumab costs around $960, while the standard 240 mg dose amounts to approximately $5,780 [15]. These prices are significantly higher than the Mexican median monthly household income of $1,390 and even exceed the top-decile income of $4,220 [16]. Pharmacoeconomic analyses suggest that dose-optimisation strategies could yield substantial cost savings without compromising outcomes [17, 18], and large-scale analyses in high-income settings, such as Medicare Part B in the United States, have highlighted the substantial and growing economic impact of immunotherapy use [19].

Half of the respondents voiced concerns regarding efficacy and safety as barriers to implementation. Pharmacokinetic and pharmacodynamic studies show PD-1 receptor saturation at doses as low as 0.1–0.3 mg/kg, suggesting that conventional dosing may exceed the minimum effective dose [20, 21]. Emerging clinical evidence from early-phase trials and retrospective studies supports a similar efficacy between low and standard-dose ICI across several indications. For example, in HNSCC, a randomised trial demonstrated improved overall survival with the addition of nivolumab at 20 mg every 3 weeks to metronomic chemotherapy, without increasing grade ≥3 adverse events [9] and similar findings have been observed in hematologic malignancies [22]. A recent systematic review concluded that low-dose PD-(L)1 inhibitor strategies often preserve efficacy and safety while improving affordability [23]. Notably, nivolumab was the most frequently investigated and prescribed low-dose ICI in both the literature and our survey, suggesting alignment between available evidence and clinical practice.

The concept of dose reduction in immunotherapy differs from cytotoxic chemotherapy, as ICI do not have a defined maximum tolerated dose and may rely more on sustained receptor occupancy than on cumulative exposure [20, 24]. In a prospective study comparing nivolumab 3 mg/kg with 40 mg flat dosing, pharmacokinetic parameters were broadly similar despite lower overall exposure [25]. Nevertheless, in the absence of randomised non-inferiority trials, a theoretical risk remains that subtherapeutic dosing could promote immune escape or resistance [26]. 

Confidence in the efficacy of low-dose ICI was lower in the curative than in the palliative setting, reflecting the scarcity of high-quality evidence in early-stage cancer. The risk-benefit assessment of low-dose ICI from both the patient and physician perspectives may also differ in this scenario, and discussion with patients about alternative dosing regimens to optimise different aspects of quality of life [27], including financial toxicity, could provide additional information on patient acceptability. Studies such as PLANeT, a phase II randomised study assessing low-dose pembrolizumab in the neoadjuvant setting for triple-negative breast cancer that reported pathological complete response rates similar to those obtained with standard dosing [28] will contribute valuable information in this regard.

Only about 20% of respondents considered that they had sufficient information to support implementation of low-dose ICI, raising the question of what type of evidence might shift practice. Although an increasing number of prospective trials are available, most compare low-dose ICI to no immunotherapy rather than to standard dosing. While non-inferiority trials would provide more definitive evidence, they are often not feasible due to cost, sample size requirements and limited industry interest [29]. Smaller trials including robust clinical outcomes and pharmacokinetic and pharmacodynamic information could provide evidence indicating near-equivalence and build confidence [30]. Public healthcare payers, who finance most healthcare systems in LMICs [31], have a clear incentive to reduce healthcare costs while preserving efficacy, so funding such pharmacoeconomic studies could be highly beneficial. A recent example is the SONIA trial in advanced breast cancer, which demonstrated non-inferiority and reduced costs by €27,078 per patient [32].

Physicians in both LMICs and high-income countries may remain reluctant to de-escalate treatment despite growing evidence supporting the clinical efficacy and potential economic benefits of low-dose strategies. For instance, meta-analyses have demonstrated non-inferiority and improved safety of 6 versus 12 months of adjuvant trastuzumab [33, 34]; however, this approach is not endorsed in the National Comprehensive Cancer Network (NCCN) Guidelines [35], while the European Society for Medical Oncology Guidelines only mention it as an option in resource-constrained settings [36]. Similarly, the Mexican Consensus for Breast Cancer does not recommend the shorter treatment duration, citing the absence of improved outcomes, rather than considering non-inferiority as a sufficient threshold for de-escalation [37].

Around one-third of respondents reported system-level barriers, including local restrictions and limitations on insurance approval. Integrating low-dose ICI options into national formularies and clinical guidelines could legitimise their use and facilitate coverage. For example, randomised non-inferiority data support the use of abiraterone 250 mg/day with food as an alternative to the standard 1,000 mg fasting regimen in metastatic castration-resistant prostate cancer [38], yet NCCN guidelines restrict this option for patients who cannot afford or will not take the standard dose [39]. When evidence supports non-inferiority, restricting dose-optimised regimens to those with financial constraints risks inadvertently perpetuating inequities in standards of care. Regulatory authorities and professional societies could issue provisional guidance on alternative dosing when access is otherwise unattainable, coupled with systematic pharmacovigilance to ensure safety.

Our study has several limitations. We observed a high rate of early attrition, which may introduce response bias, as respondents who completed the survey may differ from those who exited early. Because most non-completers provided only minimal demographic data, we were unable to formally assess these differences. In addition, due to the self-report survey design, responses may have been influenced by social desirability bias. The survey did not collect detailed dosing information or distinguish between consistently reduced dosing and occasional dose reductions across cycles; therefore, cumulative dose intensity could not be assessed. We also did not collect information on tumour site, line of therapy or indication-specific coverage, given the variability in regulatory approval, institutional formularies and payer authorisation across practice settings in Mexico. The low response rate limits the generalisability of our findings. Finally, the survey captured only physician perspectives and did not include other key stakeholders such as patients, payers and policymakers; future studies should incorporate the views of these groups.

## Conclusion

Our survey shows initial evidence of practice and interest in low-dose ICI among oncologists and hematologists in Mexico, primarily motivated by cost and access considerations. However, concerns about efficacy and safety, coupled with the absence of formal guidance, remain significant barriers to implementation. In addition to generating robust clinical, pharmacologic and health-economic evidence to guide practice, incorporating low-dose approaches into clinical guidelines and health policy discussions may be key to expanding access to immunotherapy in LMICs and beyond.

## Conflicts of interest

The authors declare that they have no conflicts of interest.

## Funding

This work was supported by the Oncology Fellowship of Costs of Care (Verduzco-Aguirre).

## Author contributions

Conception and design: ES-P-D-C, RD, LV-R, AG, MR, FC, HCV-A.

Data acquisition: AM-B, HCV-A.

Data analysis and interpretation: AM-B, HCV-A.

Initial draft: AM-B, HCV-A.

Manuscript review: ES-P-D-C, RD, LV-R, AG, MR, FC.

Final approval: All authors.

## Figures and Tables

**Table 1.** Survey respondent characteristics (N = 81).

Characteristic	n (%)
Specialty	
Medical oncology	63 (77.8%)
Hematology	18 (22.2%)
Gender	
Male	46 (56.8%)
Female	32 (39.5%)
Non-binary	2 (2.5%)
Missing	1 (1.2%)
Age (mean, SD)	44.2 ± 11.1
Years in practice (median (IQR))	9.5 (4–16.8)
Type of practice	
Public only	11 (13.6%)
Private only	23 (28.4%)
Public and private	47 (58.0%)
Number of patients treated with immunotherapy in last 12 months (median (IQR))	15 (5–27.5)
Proportion of patients who pay out-of-pocket for immunotherapy (median (IQR))	15% (5–30)
IQR: interquartile range; SD: standard deviation	

## Appendix

### Original survey in Spanish

Le invitamos a participar en una encuesta con la intención de entender el estado actual del uso de inmunoterapia con inhibidores de punto de control en México. Esta encuesta está dirigida a oncólogos médicos y hematólogos que radican en México.

La información que usted brinde será confidencial. Este estudio está aprobado por el Comité de Ética del Instituto Nacional de Ciencias Médicas y Nutrición Salvador Zubirán (HEM-4955-24-24-1).

1. ¿Cuál es su especialidad?
  a. Oncología médica
  b. Hematología
  c. Otra – esta opción termina la encuesta
2. ¿Qué edad tiene?
3. ¿Cuál es su género?
  a. Femenino
  b. Masculino
  c. No binario
  d. Prefiero no contestar
4. ¿En qué estado de la república tiene su práctica clínica principal?
5. ¿Por cuántos años ha practicado su especialidad?
6. ¿Qué tipo de práctica tiene? Seleccione todas las que apliquen
  a. Pública
  b. Privada
7. ¿Aproximadamente cuántos pacientes ha tratado con inmunoterapia (inhibidores de puntos de control) en el último año?
8. ¿En qué tipo de tumoures ha utilizado inmunoterapia en el último año? Seleccione todos los que apliquen
  a. Cáncer de mama
  b. Cáncer de pulmón
  c. Cáncer colorrectal
  d. Cáncer de estómago
  e. Hepatocarcinoma
  f. Cáncer cervicouterino
  g. Cáncer de esófago
  h. Melanoma
  i. Cáncer de vejiga/urotelial
  j. Linfoma de Hogdkin
  k. Linfoma no Hodgkin
  l. Cáncer renal
  m. Cáncer de endometrio
  n. Cáncer de cabeza y cuello
  o. Mesotelioma
  p. Otros _________________
9. ¿Ha experimentado dificultades en la adquisición o disponibilidad de inmunoterapia en su práctica clínica?
  a. Sí
  b. No
10. ¿Qué dificultades ha experimentado para la prescripción de inmunoterapia? Seleccione todas las que apliquen
  a. Restricciones regulatorias (por ejemplo, limitación en indicaciones aprobadas)
  b. Disponibilidad de los medicamentos
  c. Limitaciones geográficas (disponibilidad desigual en áreas específicas)
  d. Problemas económicos (falta de cobertura financiera, costos elevados que afectan la accesibilidad)
  e. Otras _________________
11. ¿Qué porcentaje de pacientes cubre el costo de tratamiento con inmunoterapia en su práctica clínica?
12. ¿Ha utilizado usted la inmunoterapia en dosis bajas en su práctica clínica?
  Dosis bajas: dosis menor a la dosis fija o por peso aprobada para los inhibidores de punto de control
  a. Sí
  b. No
13. ¿Aproximadamente cuántos pacientes ha tratado con dosis bajas en el último año?
14. ¿Con qué medicamentos de inmunoterapia ha utilizado dosis bajas? Seleccione todos las que apliquen
  a. Atezolizumab
  b. Durvalumab
  c. Ipilimumab
  d. Nivolumab
  e. Pembrolizumab
15. ¿Qué razones ha tenido para utilizar inmunoterapia a dosis bajas? Seleccione todas las que apliquen
  a. Reducción de costos para un paciente individual
  b. En el contexto de programas de compartición de frascos entre pacientes (reducir desperdicio de medicamento)
  c. Mejorar la tolerancia (minimizar efectos secundarios al tratamiento)
  d. Inclusión en ensayos clínicos (participar en estudios que evalúen la eficacia de dosis bajas de inmunoterapia)
  e. Otra _______________
  f. No he necesitado utilizar dosis bajas en mi práctica
16. ¿Qué barreras considera que existen para el uso de la inmunoterapia en dosis bajas? (Seleccionar todas las que correspondan)
  a. Limitaciones regulatorias de la institución
  b. Limitaciones de aprobación por seguros de gastos médicos.
  c. Preocupaciones de usted como médico tratante sobre la eficacia y/o seguridad
  d. Preocupaciones de los pacientes sobre la eficacia y/o seguridad.
  e. Falta de conocimiento sobre protocolos de dosificación alterna
  f. Problemas logísticos (por ejemplo, para compartir frascos entre varios pacientes)
  g. Otras _______________
17. ¿Cuál es su nivel de confianza en la eficacia de la inmunoterapia en dosis bajas como tratamiento en pacientes con cáncer avanzado (es decir, en un escenario con intención paliativa)?
  a. No confío en su eficacia
  b. Poca confianza en la eficacia
  c. Mucha confianza en la eficacia
  d. Confío completamente en la eficacia
18. ¿Cuál es su nivel de confianza en la eficacia de la inmunoterapia en dosis bajas como tratamiento en pacientes con cáncer en etapas tempranas (es decir, en un escenario con intención curativa)?
  a. No confío en su eficacia
  b. Poca confianza en la eficacia
  c. Mucha confianza en la eficacia
  d. Confío completamente en la eficacia
19. ¿Considera que hay suficiente información y recursos disponibles para apoyar la implementación de la inmunoterapia en dosis bajas en México?
  a. Sí
  b. No
20. ¿Cuáles considera que son los beneficios potenciales de utilizar dosis bajas de inmunoterapia en la práctica clínica? Seleccione todos los que considere.
  a. Reducción de la toxicidad y los efectos adversos
  b. Reducción de la carga financiera para los pacientes
  c. Reducción de la carga financiera para el sistema de salud
  d. Mayor flexibilidad en la administración del tratamiento
  e. Otro __________
21. ¿Cuáles considera que son los riesgos potenciales de utilizar dosis bajas de inmunoterapia en la práctica clínica? Seleccione todos los que considere.
  a. Menor eficacia terapéutica a corto plazo
  b. Menor eficacia terapéutica a largo plazo
  c. Problemas en la relación médico-paciente
  d. Otro __________
22. ¿Le interesaría participar en un estudio retrospectivo para evaluar la eficacia de la inmunoterapia en dosis bajas en pacientes con cáncer en México?
  a. Sí – se solicita correo electrónico
  b. No

### Translated survey in English

You are invited to participate in a survey designed to understand the current use of ICIs in Mexico. This survey is directed to medical oncologists and hematologists practicing in Mexico.

All information provided will remain confidential. This study was approved by the Ethics Committee of the Instituto Nacional de Ciencias Médicas y Nutrición Salvador Zubirán (HEM-4955-24-24-1).

1. What is your specialty?
  a. Medical oncology
  b. Hematology
  c. Other – this option ends the survey
2. What is your age?
3. What is your gender?
  a. Female
  b. Male
  c. Non-binary
  d. Prefer not to answer
4. In which Mexican state is your main clinical practice located?
5. For how many years have you practiced your specialty?
6. What type of practice do you have? Select all that apply.
  a. Public
  b. Private
7. Approximately how many patients have you treated with immunotherapy (ICI) in the past year?
8. For which tumour types have you used immunotherapy in the past year? Select all that apply.
  a. Breast cancer
  b. Lung cancer
  c. Colorectal cancer
  d. Gastric cancer
  e. Hepatocellular carcinoma
  f. Cervical cancer
  g. Esophageal cancer
  h. Melanoma
  i. Bladder/urothelial cancer
  j. Hodgkin lymphoma
  k. Non-Hodgkin lymphoma
  l. Renal cancer
  m. Endometrial cancer
  n. Head and neck cancer
  o. Mesothelioma
  p. Other: _____________
9. Have you experienced any difficulties obtaining or accessing immunotherapy in your clinical practice?
  a. Yes
  b. No
10. What barriers have you encountered when prescribing immunotherapy? Select all that apply.
  a. Regulatory restrictions (e.g., limited approved indications)
  b. Drug availability
  c. Geographic limitations (unequal availability across regions)
  d. Economic barriers (lack of coverage, high costs limiting access)
  e. Other: _____________
11. What proportion of your patients cover the cost of immunotherapy treatment out of pocket?
12. Have you used low-dose immunotherapy in your clinical practice?
  Low dose: any dose lower than the fixed or weight-based dose approved for checkpoint inhibitors.
  a. Yes
  b. No
13. Approximately how many patients have you treated with low-dose immunotherapy in the past year?
14. For which immunotherapy agents have you used low-dose regimens? Select all that apply.
  a. Atezolizumab
  b. Durvalumab
  c. Ipilimumab
  d. Nivolumab
  e. Pembrolizumab
15. What were your reasons for using low-dose immunotherapy? Select all that apply.
  a. To reduce costs for an individual patient
  b. Participation in vial-sharing programs (to minimise drug waste)
  c. To improve tolerability (reduce treatment-related adverse effects)
  d. Use within a clinical trial evaluating low-dose immunotherapy
  e. Other: _____________
  f. I have not needed to use low doses in my practice
16. What barriers do you perceive to using low-dose immunotherapy? Select all that apply.
  a. Institutional regulatory limitations
  b. Restrictions from insurance providers
  c. Physician concerns regarding efficacy and/or safety
  d. Patient concerns regarding efficacy and/or safety
  e. Lack of knowledge about alternative dosing protocols
  f. Logistical challenges (e.g., vial sharing across patients)
  g. Other: _____________
17. How confident are you in the efficacy of low-dose immunotherapy for patients with advanced cancer (i.e., palliative intent)?
  a. No confidence in its efficacy
  b. Low confidence in its efficacy
  c. Moderate confidence in its efficacy
  d. Complete confidence in its efficacy
18. How confident are you in the efficacy of low-dose immunotherapy for patients with early-stage cancer (i.e., curative intent)?
  a. No confidence in its efficacy
  b. Low confidence in its efficacy
  c. Moderate confidence in its efficacy
  d. Complete confidence in its efficacy
19. Do you believe there is sufficient information and resources available to support the implementation of low-dose immunotherapy in Mexico?
  a. Yes
  b. No
20. What do you consider to be the potential benefits of using low-dose immunotherapy in clinical practice? Select all that apply.
  a. Reduced toxicity and adverse effects
  b. Reduced financial burden for patients
  c. Reduced financial burden for the health system
  d. Greater flexibility in treatment administration
  e. Other: _____________
21. What do you consider to be the potential risks of using low-dose immunotherapy in clinical practice? Select all that apply.
  a. Reduced short-term therapeutic efficacy
  b. Reduced long-term therapeutic efficacy
  c. Challenges in the physician–patient relationship
  d. Other: _____________
22. Would you be interested in participating in a retrospective study to evaluate the efficacy of low-dose immunotherapy in patients with cancer in Mexico?
  a. Yes – please provide your email address
  b. No

## References

[1] Wei J, Li W, Zhang P (2024). Current trends in sensitizing immune checkpoint inhibitors for cancer treatment. Mol Cancer.

[2] Shiravand Y, Khodadadi F, Kashani SMA (2022). Immune checkpoint inhibitors in cancer therapy. Curr Oncol.

[3] Gerson R, Zatarain-Barrón ZL, Blanco C (2019). Access to lung cancer therapy in the Mexican population: opportunities for reducing inequity within the health system. Salud Publica Mex.

[4] Abrams HR, Durbin S, Huang CX (2021). Financial toxicity in cancer care: origins, impact, and solutions. Transl Behav Med.

[5] Gómez-Dantés O, Flamand L, Cerecero-García D (2023). Origin, impacts, and potential solutions to the fragmentation of the Mexican health system: a consultation with key actors. Health Res Policy Syst.

[6] Moye-Holz D, Soria Saucedo R, Van Dijk JP (2018). Access to innovative cancer medicines in a middle-income country - the case of Mexico. J Pharm Policy Pract.

[7] Go AE, Ho FDV, Feliciano EJG (2025). Barriers to immune checkpoint inhibitor access for patients with cancer in Southeast Asia: challenges and policy implications. JCO Glob Oncol.

[8] Jiang M, Hu Y, Lin G (2022). Dosing regimens of immune checkpoint inhibitors: attempts at lower dose, less frequency, shorter course. Front Oncol.

[9] Patil VM, Noronha V, Menon N (2023). Low-dose immunotherapy in head and neck cancer: a randomized study. J Clin Oncol.

[10] Trikha M, Sarkar L, Dhanawat A (2024). Performance of low-dose immunotherapy and standard-dose immunotherapy in microsatellite instability-high metastatic colorectal cancer: real-world data (CLouD-High Study). JCO Glob Oncol.

[11] Yoo SH, Keam B, Kim M (2018). Low-dose nivolumab can be effective in non-small cell lung cancer: alternative option for financial toxicity. ESMO Open.

[12] Harris PA, Taylor R, Minor BL (2019). The REDCap consortium: building an international community of software platform partners. J Biomed Inf.

[13] Hoyek C, Zheng-Lin B, Bauernfeind JJ (2025). Overcoming financial and access barriers in global cancer care with low-dose immunotherapy: a systematic review. JCO Glob Oncol.

[14] Asociación Mexicana de Instituciones de Seguros (2023). Private health insurance in Mexico. Relevance and tendencies. [El seguro de gastos médicos en México. Relevancia y tendencias].

[15] Farmacias Especializadas (2025). Catálogo. https://www.farmaciasespecializadas.com/catalogsearch/result/?q=nivolumab].

[16] Instituto Nacional de Geografía y Estadística (2025). Household income and spending [Ingresos y Gastos de los Hogares]. https://www.inegi.org.mx/temas/ingresoshog/].

[17] Hall E, Zhang J, Kim EJ (2020). Economics of alternative dosing strategies for pembrolizumab and nivolumab at a single academic cancer center. Cancer Med.

[18] Courtney PT, Yip AT, Cherry DR (2021). Cost-effectiveness of nivolumab-ipilimumab combination therapy for the treatment of advanced non-small cell lung cancer. JAMA Netw Open.

[19] Tripathi S, Bhanushali C, Majmundar V (2025). The economics of cancer immunotherapy: a five-year Medicare B expenditure analysis of checkpoint inhibitors. J Clin Oncol.

[20] Brahmer JR, Drake CG, Wollner I (2010). Phase I study of single-agent anti-programmed death-1 (MDX-1106) in refractory solid tumors: safety, clinical activity, pharmacodynamics, and immunologic correlates. J Clin Oncol.

[21] Osa A, Uenami T, Koyama S (2018). Clinical implications of monitoring nivolumab immunokinetics in non-small cell lung cancer patients. JCI Insight.

[22] Lepik KV, Fedorova LV, Kondakova EV (2020). A phase 2 study of nivolumab using a fixed dose of 40 mg (Nivo40) in patients with relapsed/refractory hodgkin lymphoma. Hemasphere.

[23] Jiménez-Labaig P, Mohamed F, Tan NJI (2025). Expanding access to cancer immunotherapy: a systematic review of low-dose PD-(L)1 inhibitor strategies. Eur J Cancer.

[24] Postow MA, Sidlow R, Hellmann MD (2018). Immune-related adverse events associated with immune checkpoint blockade. N Engl J Med.

[25] Gandhi KA, Shirsat A, Hj SK (2024). Pharmacokinetics and clinical outcomes of low-dose nivolumab relative to conventional dose in patients with advanced cancer. Cancer Chemother Pharmacol.

[26] Sharma P, Hu-Lieskovan S, Wargo JA (2017). Primary, adaptive, and acquired resistance to cancer immunotherapy. Cell.

[27] Loeser A, Kim JS, Peppercorn J (2024). The right dose: results of a patient advocate-led survey of individuals with metastatic breast cancer regarding treatment-related side effects and views about dosage assessment to optimize quality of life. JCO Oncol Pract.

[28] Arora A, Bhaskarane H, Tansir G (2025). A phase II, randomized, open-label study to evaluate low-dose pembrolizumab plus chemotherapy versus chemotherapy as neoadjuvant therapy for localized triple-negative breast cancer (TNBC) (PLANeT trial—Pembrolizumab Low dose in Addition to NACT in TNBC). Ann Oncol.

[29] Tannock IF, Buyse M, De Backer M (2024). The tyranny of non-inferiority trials. Lancet Oncol.

[30] Tannock IF, Ratain MJ, Goldstein DA (2021). Near-equivalence: generating evidence to support alternative cost-effective treatments. J Clin Oncol.

[31] Gómez-Dantés O, Serván-Mori E, Cerecero D (2024). Mexico's health system,. Salud Publica Mex.

[32] Sonke GS, Van Ommen-nijhof A, Wortelboer N (2024). Early versus deferred use of CDK4/6 inhibitors in advanced breast cancer. Nature.

[33] Earl HM, Hiller L, Dunn JA (2025). Reduced duration adjuvant trastuzumab in the treatment of patients with HER2-positive breast cancer: a meta-analysis of randomised controlled non-inferiority trials including IPD data. BMJ Oncol.

[34] Eiger D, Franzoi MA, Pondé N (2020). Cardiotoxicity of trastuzumab given for 12 months compared to shorter treatment periods: a systematic review and meta-analysis of six clinical trials. ESMO Open.

[35] National Comprehensive Cancer Network (2025). NCCN clinical practice guidelines in oncology (NCCN Guidelines®). Breast Cancer.

[36] Loibl S, Andre F, Bachelot T (2024). Early breast cancer: ESMO clinical practice guideline for diagnosis, treatment and follow-up. Ann Oncol.

[37] Consenso Mexicano sobre Diagnóstico y Tratamiento del Cáncer Mamario (2025).

[38] Szmulewitz RZ, Peer CJ, Ibraheem A (2018). Prospective international randomized phase II study of low-dose abiraterone with food versus standard dose abiraterone in castration-resistant prostate cancer. J Clin Oncol.

[39] National Comprehensive Cancer Network (2025). NCCN clinical practice guidelines in oncology (NCCN Guidelines®). Prostate Cancer.

